# Influence of interpersonal relationship on subjective well-being of college students: The mediating role of psychological capital

**DOI:** 10.1371/journal.pone.0293198

**Published:** 2024-09-20

**Authors:** Jinyong Zhang, Shouying Zhao, Huaiqing Deng, Chuan Yuan, Zhi Yang

**Affiliations:** 1 College of education science, Guizhou Education University, Guiyang, China; 2 School of Psychology, Guizhou Normal University, Guiyang, China; Shenzhen University, CHINA

## Abstract

**Background:**

Nowadays, the contradiction between the rapid improvement of people’s material living standard and the loss of spiritual pursuit or the increase of pressure is becoming more and more serious.With the impact of the new corona-virus pandemic, the employment situation of college students is more severe. This leads to the growth of psychological problems and alienation behavior of college students. In the promoting positive psychology and enhancing the happiness of the whole people, the relationship between interpersonal relationship and subjective well-being of college students is an interesting and valuable research topic.

**Methods:**

The structural model with AMOS21.0 was used to verify the mediating effect of psychological capital between interpersonal relationship distress and SWB from the survey data of 673 college students.

**Results:**

The interpersonal distress has a negatively correlated with psychological capital and SWB, while psychological capital has a positively correlated with SWB. Psychological capital has Partial mediating effect between interpersonal distress and SWB, and the indirect effect accounts for 57% of the total effect.

**Conclusion:**

This study indicates that schools should pay attention to education and intervention in psychological capital, except for strengthening knowledge and skill training of college students in how to communicate with others.

## Introduction

In recent years, the contradiction between the rapid improvement of people’s material living standards and the loss of spiritual pursuit or increased pressure has become increasingly prominent. The employment situation of college students is more severer for the impact of the COVID-19. This leads to the growth of college students’ psychological problems and alienation behavior [1–3]. In the advocating positive psychology and improving the sense of well-being of the whole people, college students’ Subjective well-being (SWB) become an interesting and valuable topic. At the same time, due to the long-term impact of the new corona-virus pandemic, the college delay the opening time and carry out online teaching. The activities and exercises of college students’ were greatly limited, resulting in some problems in college students’ interpersonal relationships.

SWB, as the ultimate goal of an individual’s life, becomes a comprehensive psychological index to measure the quality of individual life [4]. Downie thinks that health must also include both "true well-being" and "physical fitness" and be able to adjust to the imbalance between positive and negative health [5]. This shows that happiness is an integral part of mental health. Studies have shown that SWB is beneficial to health and longevity, supportive social relationships and resilience [6]. It has specific positive significance for specific groups. SWB plays a promoting role between Muslim religious beliefs and healthy lifestyles [7]. Couples with subjective happiness pessimism are also easy to establish healthy interpersonal relationships [8]. In the hotel industry, it affects employee knowledge sharing and willingness to leave [9,10]; It helps to improve the consumption of saturated fat [11]. Even it can reduce college teachers’ network teaching anxiety [12]. Generally speaking, positive SWB is beneficial in life activities. Therefore, a college student with a high SWB experience must be conducive to the healthy growth and creative stimulation of college students.

Many factors affect SWB, such as heredity [13], basic material needs and psychological needs [14], income [15], temperament [6], partnership [16], patience [17], character optimism and resilience [18]. These factors can be divided into two categories: personality and situation. The personality-situation interaction theory shows that the interaction between personality and environment affects SWB [19]. Interpersonal relationship is an important part of social situation, and psychological capital is the main element of personality traits. Psychological capital is a positive psychological element formed in the process of individual growth and development, including self-efficacy, optimism, resilience and hope [20]. This study aims to explore the relationship between interpersonal relationship, psychological capital and SWB of college students and its internal mechanism.The purpose of this study is to provide a theoretical basis for the improvement of college students ’ happiness education.

### Theoretical basis

Personality-situation interaction theory holds that the interaction between personality and environment affects SWB [19]. Here, psychological capital and interpersonal relationship are selected to represent personality and situation respectively to study the influence on SWB. Erickson’s personality development theory tells us that college students are in the development stage of intimacy and loneliness. Positive traits pays special attention to the positive quality of individuals, which is the source of strength for individuals to cope with crises and obtain happiness [21]. SWB is the emotional component of positive psychology and the core of positive psychology. Psychological capital is the foundation and premise of SWB.

### Interpersonal relationship and SWB

An individual’s interpersonal relationship is harmonious, his SWB experience will be stronger. It can affect the formation of a positive personality. On the contrary, an individual has been plagued by interpersonal relationships, it will reduce his SWB. Zhang XX study shows that parent-child, mates, and teacher-student relationships can positively predict SWB in junior and high school students [22]. Zhao HL studied results showed that emotional support had the greatest predictive effect on SWB [23]. Children’s interpersonal relationship is associated with SWB satisfaction [24]. Good interpersonal relationships have a significant predictive effect on SWB [25].

### Psychological capital and SWB

Psychological capital refers to a positive state of mind, which exists in the individual in the process of growth and development. It can be acquired and developed through training. Psychological capital has a promoting effect on their mental health and SWB. Specifically, students’ well-being and psychological capital is significantly positive correlation [26]. Psychological capital also has a strong predictive effect on the SWB in the old people [27,28].

### Interpersonal relationship and psychological capital

Self-expansion theory holds that individuals seek to obtain resources, such as positive interpersonal relationships, to promote growth and achievement [29]. Psychological capital can correctly guide interpersonal relationship [30]. There is an approximately linear correlation between psychological capital and interpersonal communication [31].

Studies have shown that the peer relationship of migrant adolescents can also indirectly predict subjective well-being through academic burnout or academic engagement [32]. Based on the above research results, this study intends to investigate the relationship between interpersonal relationship, psychological capital and SWB, and explore the intermediate mechanism between interpersonal relationship and SWB from the perspective of personality and psychological capital. This study proposes the following research hypotheses:

1. There is a significant negative correlation between interpersonal distress and psychological capital.
2. There is a significant positive correlation between psychological capital and SWB.
3. Interpersonal relationship distress and psychological capital have a significant predictive effect on SWB.
4. Psychological capital plays an intermediary role in the influence of interpersonal relationships on SWB.

**Fig 1:**
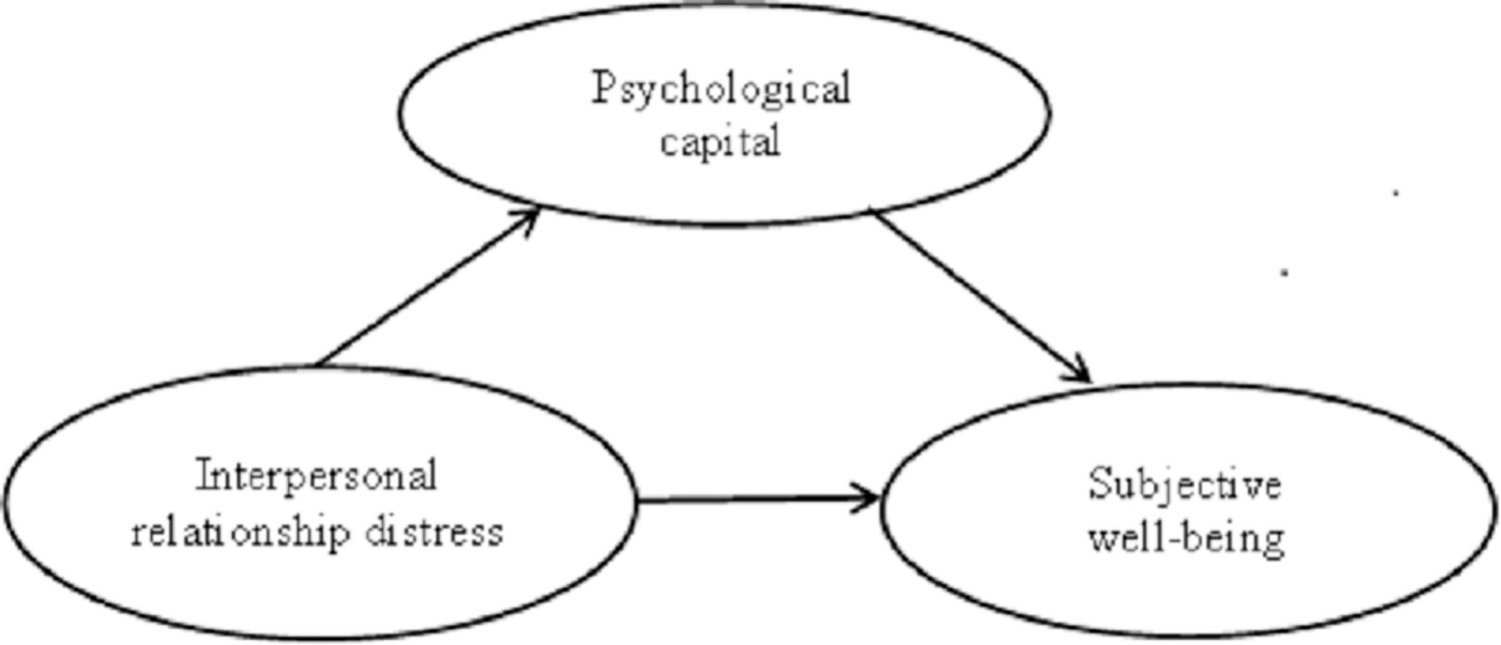
Mediation effect path diagram of psychological capital.

The mediation structure model is shown in **Fig 1**.

## Materials and methods

### Ethics statement

This study was approved by the Ethics Committee of School of Psychology, Guizhou Normal University (GZNU-PSY-2022-003). Before giving out the questionnaire, the researcher read out the instruction to the participants, including the words of thanks, questionnaire Guideline, the research purpose, and the right of participation, which can be terminated halfway. All subjects signed informed consent before investigation.

### Participants

The investigation time was in April 2022. There were 700 undergraduate students enrolled in this survey with the support of faculty members at three universities in Guiyang. The participants were psychologically healthy college students. After providing oral guidance and explaining the purpose of the study, the students willing to participate in the experiment were required to provide informed consent form after their data will be confidential. If any missing information was found when retrieving the questionnaire, participants were required to refill in. Perfunctory and problematic questionnaires were excluded, such as the same option in selection of multiple consecutive items. Finally, 673 questionnaires were valid, with an effective rate of 96.1%. The subjects included male and female, Han and ethnic minorities, and freshmen to juniors (Table 1).

**Table 1:** Participants’ sociodemographic characteristics (N = 673).

Variables		Mean	SD
Age		19.92	1.29
		Number	Ratio%
Sex	Male	254	37.7
	Female	419	62.3
Nation	Han nationality	398	59.1
	Ethnic Minorities	275	40.9
Grade	Freshmen	245	36.4
	Sophomores	214	31.8
	Juniors	214	31.8

### Informed consent

All participants in this study orally agreed to take part in the experiment.

### Research tools

#### Interpersonal relationship comprehensive diagnosis scale

The scale was compiled by Deng RC. There are 28 questions within four dimensions: interpersonal communication problems, interpersonal friendship problems, interpersonal relationship problems, heterosexual communication problems [33]. Each dimension have 7 questions, using 2-level scoring, no reverse scoring, choose ‘Yes’ get 1 point, ‘No’ get 0 points. The scores ranged from 0 to 8, indicate good interpersonal skills. The scores ranged from 9 to 14, indicate a certain degree of trouble in communicating with people and the interpersonal skills are average. The scores are between 15 and 28, indicating that there is a great deal of trouble in getting along with people and a serious lack of interpersonal skills. This scale has good reliability and validity. In this study, the overall scale showed high internal consistency (Cronbach’s = 0.86). and the internal consistency coefficients of the four dimensions were also higher (Cronbach’s = 0.60~0.72). The AMOS21.0 software was used to construct structural equation with interpersonal communication distress, interpersonal friendship distress, interpersonal relationship distress and heterosexual interaction distress as latent variables, and their corresponding topics as explicit variables. The results of confirmatory analysis were as follows: x^2^/df = 3.25, GFI = 0.92, AGFI = 0.91, CFI = 0.78, RMSEA = 0.058.

#### Psychological capital scale

The scale was compiled by Luthans and revised by Song, including 16 questions and four dimensions: self-efficacy, hope, optimism, and resilience [34]. The reliability of the revised scale was 0.89, and the retest reliability was 0.70. The reliability of each sub-scale ranges from 0.73 to 0.81, and the retest reliability ranges from 0.50 to 0.62. Likert 5 scale was used for positive scoring, the “Very inconsistent” to “Very consistent” were recorded as 1 ~ 5 points, no reverse scoring. The higher the score is, the better the level of psychological capital. In this study, the psychological capital scale indicated high internal consistency (Cronbach’s = 0.88). and The internal consistency coefficients of the four dimensions are also higher (Cronbach’s = 0.65~0.81). Taking self-efficacy, hope, optimism and resilience as latent variables and their corresponding topics as explicit variables, the structural equation was constructed by AMOS21.0 software.The results of confirmatory analysis were: X^*2*^/DF = 2.67, GFI = 0.95, AGFI = 0.94, CFI = 0.96,RMSEA = 0.050.

#### SWB scale

The scale was compiled by Diener. It is consisted of three sub-scales: life satisfaction, positive emotion and negative emotion, with 19 items [4]. The scale adopt the 7-grade scoring method; each item marks 1–7 points. The total score of the scale was the sum of 19 items. The higher the total score, the better the SWB. In this study, the overall scale indicated high internal consistency (Cronbach’s = 0.81). and The internal consistency coefficients of the three dimensions are also higher (Cronbach’s = 0.80~0.87). Taking life satisfaction, positive emotions and negative emotions as latent variables, and their corresponding topics as explicit variables, the structural equation was constructed by using AMOS21.0 software. The results of confirmatory analysis were: X^*2*^/DF = 6.68, NFI = 0.83, IFI = 0.85, CFI = 0.85, RMSEA = 0.092.

### Statistical analysis

The software SPSS21.0 was used for data analysis. Pearson correlation analysis was carried out on interpersonal relationship, psychological capital and SWB. The regression analysis was conducted with SWB as the dependent variable, interpersonal distress and psychological capital as predictive variables. Finally, according to the requirements of testing the mediating effect, all independent variables are standardized firstly [35]. Taking interpersonal relationship as independent variable, psychological capital as intermediary variable and SWB as dependent variable, the intermediary structure model was constructed using AMOS21.0. According to the model fitting index and Bootstrap test results, the latter is based on 5000 repeated sampling, and ***95%*** of the deviation correction confidence interval is selected. If the indirect effect path interval does not contain 0, this shows that psychological capital is the intermediary effect of interpersonal relationship on SWB. Otherwise, there is no intermediary effect.

## Results

### Common bias method

To prevent common method bias, corresponding controls were made in questionnaire setting and implementation, such as anonymous responses, differentiated scale scoring parties and reverse question setting. The ***Harman*** one-way test was used to test common method bias. All items were subjected to dimensional reduction analysis. The results showed that the total number of factors with unrotated eigenvalues greater than 1 was 13, and the variance explained by the first factor was 16.38, which was less than the critical criterion of 30%, which indicated that the common method bias was not significant [2,36].

### Comparison of socio-demographic variables of interpersonal relationships, psychological capital and SWB

The independent sample t-test showed that there was no significant difference between male and female college students in interpersonal distress, psychological capital and SWB; but ethnic minorities was significantly higher than Han Chinese in the mean value of psychological capital. The one-way ANOVA results showed that there were no significant difference between the three grades (Table 2). so it was not necessary to control for demographic variables.

**Table 2:** Comparison of differences in socio-demographic variables (M±SD).

Variables	Category	Interpersonal distress	Psychological capital	SWB
Sex	Male	55.47+9.16	9.40+6.09	83.40+12.52
	Female	54.23+8.05	9.14+5.25	84.42+13.36
t		1.78	0.57	-0.99
Nation	Han nationality	54.79+8.82	8.87+5.53	84.81+13.08
	Ethnic Minorities	54.57+8.04	9.77+5.63	82.92+12.95
t		0.35	-2.06^*^	1.85
Grade	Freshmen	53.76+7.87	8.77+5.09	84.70+13.55
	Sophomores	55.43+9.30	9.36+5.77	84.38+13.34
	Juniors	55.06+8.31	9.65+5.90	82.94+12.13
F		2.50	1.52	1.14

### Descriptive statistics and correlation analysis of the main variables

In this study, mean and standard deviation, and Pearson product difference correlations were conducted for the relationships among interpersonal relationships, psychological capital, SWB, and their dimensions (Table 3). The average score of interpersonal problems was between 9 and 14 points, which indicate that there was a certain degree trouble of college students’ in interpersonal communication. The overall level of psychological capital was above average, and the overall level of SWB was higher. Interpersonal relationship distress was negatively correlated with psychological capital and SWB (r = -0.37, p<0.01; r = -0.48, p<0.01), while psychological capital is positively correlated with SWB (r = 0.45, p<0.01). The findings are consistent with the first three hypotheses.

**Table 3:** Descriptive statistics and correlation analysis of interpersonal distress, psychological capital and SWB.

Contents	M	SD	1	2	3
1.Interpersonal distress	9.24	5.58	-		
2.Psychological capital	54.70	8.50	-0.366**	-	
3.SWB	84.04	13.05	-0.476**	0.445**	-

Note: *p<0.05,**p<0.01,***p<0.001, the same below.

### Regression analysis of interpersonal relationship and psychological capital on SWB

The regression analysis was conducted using the "enter" method, SWB as the dependent variable, and interpersonal distress and psychological capital as predictor variables. The fit of the regression equations for interpersonal distress and psychological capital was high (F = 196.62, p<0.001; F = 165.99, p<0.001) (Table 4). The interpersonal distress had significant negative predictive power on SWB (β = -0.476, t = -14.02, p<0.001) and psychological capital had positive predictive power on SWB (β = -0.445, t = -12.88, p<0.001). This results verified hypothesis 3.

**Table 4:** Results of regression analysis.

dependent variable	predictor variable	*R* ^*2*^	Adjusted R^*2*^	*F*	Normalization coefficient (Beta)	*t*
SWB	Interpersonal distress	22.7%	22.5%	196.62***	-0.476	-14.02***
	Psychological capital	19.8%	19.7%	165.99***	0.445	12.88***

### Relationship between interpersonal relationship and SWB: The intermediary role of psychological capital

**Fig 2:**
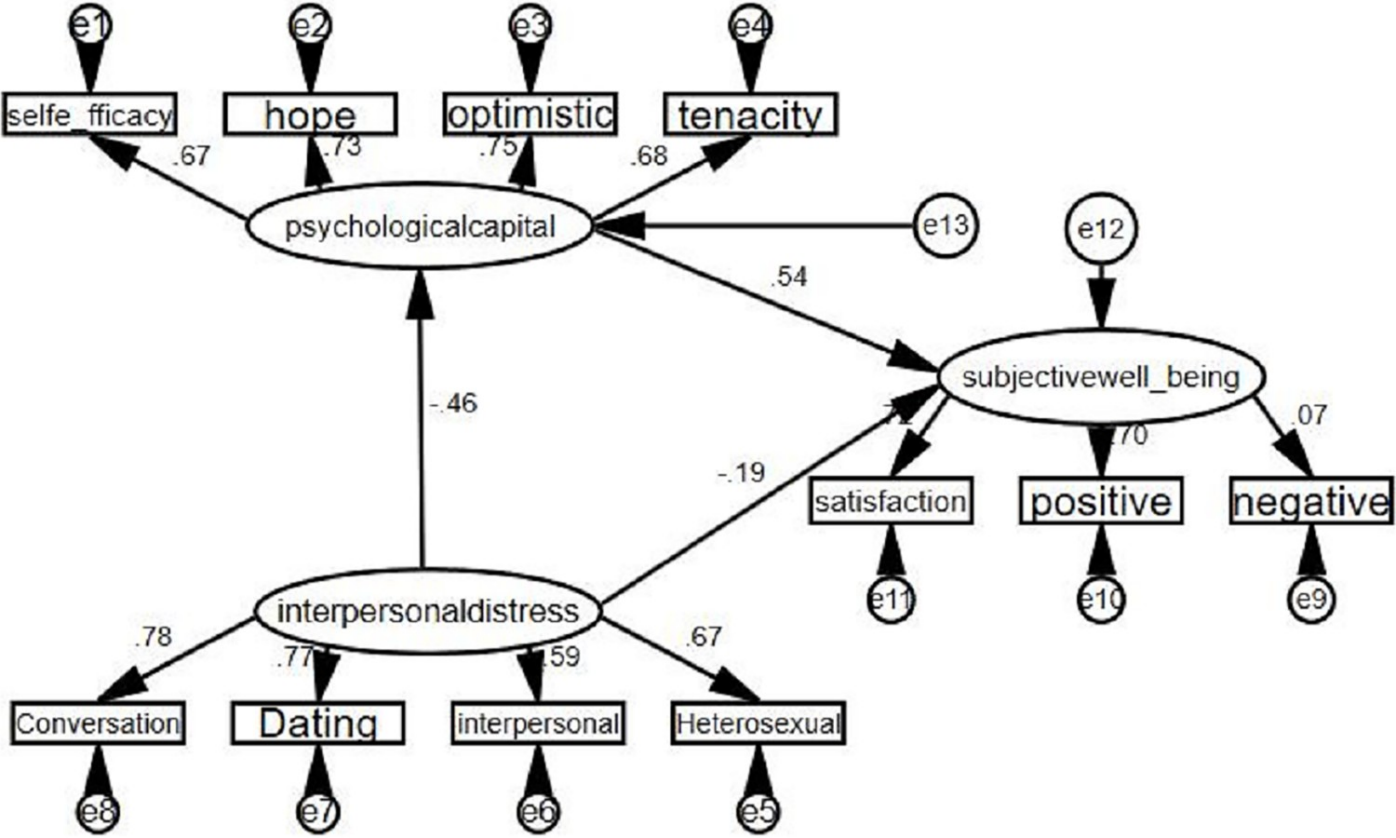
Mediating effect of psychological capital on interpersonal distress and SWB (standardized results).

We use a structural equation model to investigate the intermediary role of psychological capital、 interpersonal distress and SWB (**Fig 2**). The fitting indexes of the model are good (Table 5).

**Table 5:** Fitting index of intermediary structure equation.

df	X^2^	x^2^/df	p	RMSEA	CFI	IFI	NFI	TFI
41	248.28	6.06	< 0.01	0.09	0.91	0.91	0.89	0.88

The mediating effect of psychological capital between interpersonal distress and SWB was examined according to the bootstrapping method (Table 6). The results showed that the confidence interval for the prediction of SWB by interpersonal distress through psychological capital did not include 0 (-0.567~-0.168), after 5000 replicate samples, 95% bias-corrected confidence intervals, which indicate that the mediating effect of interpersonal distress to predict SWB through psychological capital could be obtained to hold. Meanwhile, the confidence interval of interpersonal distress directly on SWB did not include 0 (-0.095~ -0.003), indicating that there is a partial mediating effect of interpersonal distress to predict SWB through psychological capital, which accounts for about 57% of the total effect. At this point, hypothesis 4 was tested.

**Table 6:** Bootstrap test and effect value of mediation effect (N = 673).

	path	Standardized effect estimation	Effect quantity	95% confidence interval	
				lower limit	upper limit
Indirect effect	Interpersonal distress → Psychological capital → SWB	0.248	57%	-0.567	-0.168
Direct effect	International distress → SWB	-0.187	43%	-0.095	-0.003
Total effect		0.435	100%		

## Discussion

### The characteristics of interpersonal distress, psychological capital and SWB

This study showed that there are certain problems in interpersonal relationship distress among college students. Those with scores above 9 were 53.5%, which is consistent with the results of "47.8% of college students have some or serious interpersonal relationship problems" by Tan [37]. This indicates that college students lack basic interpersonal skills, and it may also be related to the restriction of their living space and activities due to the epidemic. Interpersonal distress can affects mental health [38]. Therefore, education and training in this area need to be enhanced to improve the quality of life. The psychological capital scores of college students are above the median, which indicates that college students have some positive traits and better psychological resilience. This result is consistent with the findings of Xu that "college students’ psychological capital and the development of each dimension are at an intermediate level"[39]. But, the overall SWB of college students is relatively high, which is contrary to the results of Chen [40], who thought that college students have a "moderate to low" level. The difference of the results may be related to the different measurement instruments used or the different study time. It may be also related to the low academic stress and the richness of recreational and sports activities among college students in these colleges.

### The relationship between interpersonal distress, psychological capital and SWB of college students

The correlation analysis revealed a significant negative correlation between interpersonal distress and psychological capital, which is consistent with the findings of Zhang’s [41]. This indicates that interpersonal distress is an important factor in weakening psychological capital. The interpersonal distress was negatively correlated with SWB and effectively predicted SWB, which is consistent with the findings of Liu’s that "interpersonal relationship satisfaction and SWB are both significantly positively correlated" [42]. This indicates that the poorer the interpersonal relationship, the lower happiness. Psychological capital was significantly positively correlated with SWB and positively predicted SWB, which is consistent with the findings of Xiong’s [43]. It indicates that the amount of positive traits a person possesses directly affects his level of happiness.

### The influence of college students’ interpersonal distress on SWB: The mediating effect of psychological capital

The mediation analysis found that the partial mediating role of college students’ psychological capital between interpersonal distress and SWB was statistically significant, suggesting that interpersonal distress directly affects the level of SWB and can also indirectly act on the level of SWB through psychological capital. According to Maslow’s hierarchy of needs, the need for interaction is a developmental need of individuals, especially in today’s advanced Internet communication, college students need realistic interaction space. As college students have just got rid of the monotonous and stressful learning environment in secondary school, they are bound to lack the corresponding knowledge and skills in interpersonal conversation, making friends, treating others and interacting with the opposite sex. Luthans argue that psychological capital is a state of positive psychological development that individuals exhibit during their growth and development, that contains four aspects of self-confidence or self-efficacy, hope, optimism, and resilience [34]. Both states and traits, psychological capital has positive significance for individuals. A college student who has great psychological capital tends to have one or several psychological qualities. If the individual has a high sense of self-efficacy, he will has a positive and optimistic attitude with interpersonal distress. The fact that interpersonal distress indirectly affects SWB through psychological capital supports the view of personality trait theory of well-being that individuals have positive personality traits and this personality tends to experience life in a positive way, in which happiness is generated. In conclusion, psychological capital not only plays an important role in mitigating and protecting against interpersonal distress, but also contributes to the enhancement of well-being. The psychological capital mediating effect model is further revealing the mechanism of interpersonal relationships and subjective well-being.

The individuals with high psychological capital have positive expectations about future outcomes and they show higher beliefs in dealing with challenges and difficulties that arise at work [44]. Guo found that psychological capital of knowledge employees has a significant positive effect on innovation performance [45]. Psychological capital not only affects individual performance but also indirectly affects group work efficiency. Zhang’s study found that psychological capital is beneficial to improve organizational performance [46]. Also psychological capital has a positive predictive effect on mental health [47]. In conclusion, psychological capital, as a positive psychological resource for individuals, facilitates individuals to face the difficulties and setbacks, and promotes their healthy growth. This also validates again the mediating effect of psychological capital on SWB.

### Educational suggestions

#### Strengthen the knowledge and skills training on how to interact with others

The university is the front door to the society. Social interaction will become a compulsory course in college students life. The students should strengthen their knowledge of social interaction, especially interaction etiquette, and understand the importance of interpersonal interaction to personal growth and environmental adaptation. In recent years, due to the impact of the epidemic, universities often implement closed management, which limits the way and space of college students’ activities to a certain extent. Interpersonal communication is also affected, and even some college students have some psychological obstacles. The school should build various social platforms for college students and encourage students to participate in various after-school activities, The school should Also actively organize appropriate social science lectures or offer relevant courses to improve the humanistic and comprehensive quality of college students.

#### Education and intervention of college students’ psychological capital

Psychological capital can be developed by intervention [25]. Psychological traits are the inevitable result of the development of psychological states. Psychological capital mainly includes qualities such as self-efficacy, hope, optimism and resilience. College students should learn to self-regulate and self-reinforce. The schools should provide more opportunities for students to practice and set appropriate goals. Exercise is an effective way to enhance psychological capital. Qin found that moderate intensity physical exercise enhanced the psychological capital of college students through a group experimental intervention study [48]. These suggestions have important value for the enhancement of well-being of primary and secondary school students. Even psychological capital has a positive impact on encouraging Organizational commitment [49] and organizational citizenship behavior [50]. This proves the universal significance of enhancing psychological capital ability.

## Strengths and limitations

The advantage of this study was using the questionnaire to quickly confirm that psychological capital plays a mediating role in the influence of interpersonal relationships on SWB. But, this study took a cross-sectional approach so that the variable relationships could only be causal in a statistical sense. The longitudinal studies or experimental methods should be used for more reliable scientific conclusions in the future. In addition, the four dimensions of psychological capital can be considered to be tested separately when studying the mediating role of psychological capital in interpersonal relationship and SWB.

### Implication of this study

This study intends to investigate the relationship between interpersonal relationship, psychological capital and SWB, and explore the intermediate mechanism between interpersonal relationship and SWB from the perspective of personality and psychological capital. Further enrich the theory of subjective well-being, and at the same time, this study explores the direct and indirect factors that affect subjective well-being, providing theoretical support for improving the practical path of college students’ subjective well-being.

## Conclusion

Our research shows that psychological capital is a modifiable factor in the relationship between interpersonal distress and SWB. Psychological capital has a significant positive correlation with SWB. Specifically, on the one hand, the interpersonal distress has a negatively correlated with SWB of college students; on the other hand, due to the protective role of college students’ psychological capital, interpersonal relationships are greatly reduced, thereby enhancing their subjective well-being. Therefore, the university should not only strengthen the knowledge and skills of interpersonal communication, but also improve the psychological capital ability of college students through labor and frustration education, so as to improve the subjective well-being of college students.

## Supporting information

S1 Data (SAV)

## References

[1] Shengyao H. Mental Health Assessment of Post-00s College Students Based on Knowledge Network. Wireless Communications and Mobile Computing. 2022. 10.1155/2022/1966057.

[2] Ewing L, Hamza CA, Walsh K, Goldstein AL, Heath NL. A Qualitative In-vestigation of the Positive and Negative Impacts of the COVID-19 Pandemi-con Post-Secondary Students’Mental Health and Well-Being. Emerging Adul-thood. 2022; 10(5):1312–1327. 10.1177/21676968221121590. PMC9393400 36111320

[3] Hu XB, Yang YN, Zhang MY, et al. Present Situation and Influencing Fact-ors on Classroom Mobile Phone Dependence Syndrome in College Students. Chinese Journal of Epidemiology. 2017;38(10):1352–1357. doi: 10.3760/cma.j.issn.0254-6450.2017.10.011 29060978

[4] Liu BZ, Liang JL. A Review of the Research on Subjective Well-being of College Students in China in Recent Years. Journal of Guangdong Youth Vo-cational College. 2015; 29 (02): 69–72.

[5] Turliuc MN, Candel OS. The Relationship between Psychological Capital an-d Mental Health during the Covid-19 Pandemic:A Longitudinal Mediation Model. Journal of Health Psychology. 2021;27(8):1913–1925. doi: 10.1177/13591053211012771 33913353

[6] Diener E, Oishi S, Tay L. Advances in Subjective Well-being Research. Nat-ure Human Behaviour. 2018; 2(4):253–260. doi: 10.1038/s41562-018-0307-6 30936533

[7] Tey SE, Park MSA, Golden KJ. Religiosity and Healthy Lifestyle Behaviou-rs in Malaysian Muslims:The Mediating Role of Subjective Well-being and Self-regulation. Journal of Religion and Health. 2017;57(6):2050–2065. 10.1007/s10943-017-0420-2. 28647911

[8] Borenstein-Laurie JB, Scheier MA, Wrosch MF, Wrosch C. Examining intra- and Inter-personal Health Effects of Optimism and Pessimism: The Role of Subjective Well-being in Romantic Couples. Journal of Personality. 2022; 22(6):1–56. doi: 10.1111/jopy.12768 36017583

[9] Bhatti MH, Akram U, Bhatti MH, Rasool H, Su X. Unraveling the Effects of Ethical Leadership on Knowledge Sharing:The Mediating Roles of Subjec-tive Well-Being and Social Media in the Hotel Industry.Sustainability. 2020; 12(20):8333–8333. 10.3390/su12208333.

[10] Maslakçı A, Lütfi S, Sesen H. Moderator Role of Subjective Well-being in the Impact of COVID-19 Fear on Hotel Employees’ Intention to Leave. Jo-urnal of Human Resources in Hospitality & Tourism. 2022; 21(1):57–81. 10.1080/15332845.2022.2015232.

[11] Mullan B, Xavier K. Predicting Saturated Fat Consumption:Exploring the Role of Subjective Well-being. Psychology, Health & Medicine. 2013; 18(5):515–21. doi: 10.1080/13548506.2013.764456 23350606

[12] Zhang X, Li SQ, Wang SW. et al. Influence of Job Environment on the Online Teaching Anxiety of College Teachers in the Online Teaching Context: The Mediating Role of Subjective Well-being. Frontiers in Public Health. 2022; 10:978094–978094. doi: 10.3389/fpubh.2022.978094 36311626 PMC9614316

[13] Røysamb E, Nes RB. The Role of Genetics in Subjective Well-being. Nat-ure Human Behaviour. 2018; 3(1):3. 10.1038/s41562-018-0494-1. 30932049

[14] Tay L, Diener E. Needs and Subjective Well-being Around the World. Jour-nal of Personality and Social Psychology. 2011;101(2):354–65. doi: 10.1037/a0023779 21688922

[15] Diener E, Ng W, Harter J. et al. Wealth and Happiness across the World: Material Prosperity Predicts Life Evaluation, Whereas Psychosocial Prosperit Predicts Positive Feeling. Journal of Personality and Social Psychology. 2010;99(1):52–61. 10.1037/a0018066. 20565185

[16] Johnson MD, Neyer FJ, Finn C. Subjective Well-being across Partnerships. Journal of Family Psychology. 2020; 35(4):546–551. doi: 10.1037/fam0000793 32790465

[17] Reza F, Amir H, Kazmi S. Impact of Smartphones, Self-determination and Patience on Subjective Well-being of Bottom of Pyramid Customers. Revist-a Brasileirade Marketing. 2021; (2): 279–308. doi: 10.5585/REMARK.V20I2.17569

[18] He F, Cco R, Feng H. et al. The Impacts of Dispositional Optimism and Psychological Resilience on the Subjective Well-being of Burn Patients: a Structural Equation Modeling Analysis. PLOS ONE. 2013; 8(12): e82939. 10.1371/journal.pone.0082939. 24358241 PMC3866201

[19] Wrzus C, Wagner G, Riediger M. Personality-situation Transactions from Adolescence to Old Age.Journal of Personality and Social Psychology. 2016;110(5):782–99. doi: 10.1037/pspp0000054 26167797

[20] Natkhov T. Education,Social Capital, and Economic Development (Review of Basic Studies). Voprosy Ekonomiki. 2010; (8):112–122. 10.32609/0042-8736-2010-8-112-12221.

[21] Tabernero C, Caprara GV, Gutiérrez-Domingo T, et al. Positivity and Self-Efficacy Beliefs Explaining Health-Related Quality of Life in Cardiovascular Patients. Psicothema. 2021; 33(3):433–441. doi: 10.7334/psicothema2020.476 34297673

[22] Zhang XX, Guo HY, Lin DH. A Study on the relationship between Parent-child, Peer, Teacher-student Relationship and subjective well-being of adole-scents. Psychological Development and Education. 2019; 35(4):458–466. doi: 10.16187/j.cnki.issn1001-4918.2019.04.09

[23] Zhao HL, Chen SC. Study on the Relationship between Mental Health, So-cial Support and Subjective Well-being of Boarding Middle School Stude-nts Taking in Tibetan Residential Area of Qinghai Province as an Exampl-e. Journal of Qinghai Normal University (Philosophy and Social Sciences Edition). 2016; 38(05):155–160. doi: 10.16229/j.cnki.issn1000-5102.2016.05.028

[24] Dos Santos BR, Sarriera JC, Bedin LM. Subjective Well-Being, Life Satisf-action and Interpersonal Relationships Associated to Socio-Demographic and Contextual Variables. Applied Research in Quality of Life. 2018; 14(3):819–835. 10.1007/s11482-018-9611-625.

[25] Martínez LM, Estrada D, Prada SI. Mental Health, Interpersonal Trust and Sub-jective Well-being in a High Violence Context. SSM-Population Health.2019; (8):100423. https://www.ncbi.nlm.nih.gov/pubmed/31321278. 31321278 10.1016/j.ssmph.2019.100423 PMC6612929

[26] Peng J, Li XY. Research on the relationship between college students’ happiness and psychological capital. Journal of Inner Mongolia Normal U-niversity (Education Science Edition). 2014; 27(10):109–110.

[27] Liu LL, Guo W, Liu TT. et al. The Influence of Psychological Capital on the Subjective Well-being of the Elderly. Chinese Journal of Gerontology.2014; 34(12):3411–3413.

[28] Heinitz K, Lorenz T, Schulze D, Schorlemmer J. Positive Organizational Be-havior: Longitudinal Effects on Subjective Well-being. PLOS ONE. 2018;13(7): e0198588. 10.1371/journal.pone.0198588. 29933367 PMC6014654

[29] Park J, Uhm JP, Kim S. et al. Sport Community Involvement and Life Sa-tisfaction during COVID-19: A Moderated Mediation of Psychological Cap-ital by Distress and Generation Z. Frontiers in Psychology. 2022; 13:863610. 10.3389/fpsyg.2022.861630. PMC9048900 35496150

[30] Jiang YJ, Bai L. The Influence of Psychological Capital Construction on College Students’ Interpersonal Relationship and Subjective Well-being. Hei-longjiang Higher Education Research. 2015(09):130–132.

[31] Lu FL. The Influence of College Students’ Psychological Capital on Acade-mic Performance and Interpersonal Relationship. Journal of Kaifeng Instit-ute of Education. 2017; 37(07):161–163.

[32] Ma BB, Dai WJ, Li CN. Interpersonal Relationship and Subjective Well-be-ing of Migrant Youth Schools:the Mediating Role of Academic Burnout and Academic Engagement. China Special Education. 2019; (12):63–71

[33] Zheng RC. Psychological Diagnosis of College Students. Jinan: Shandong Education Press. 2002, 339–341.

[34] Song HF, Mao TW.Revision and Reliability and Validity Test of Mental Capital Scale in College Students. Statistics and Decision. 2012; (21):106–109. doi: 10.13546/j.cnki.tjyjc.2012.21.017

[35] Preacher KJ, Kelley K. Effect Size Measures for Mediation Models: Quan-titative Strategies for Communicating Indirect Effects. Psychol Methods. 2011; 16(2):93–115. doi: 10.1037/a0022658 21500915

[36] Aleinov I, Way MJ, Harman C, Tsigaridis K, Wolf ET, Gronoff G. Modeli-ng a Transient Secondary Pale-Lunar Atmosphere:3-D Simulations and An-alysis. Geophysical Research Letters. 2019; (5):1–19. 10.1029/2019gl082494.

[37] Tan YF, Cheng Y. Research on the Relationship between College Students’ Positive Emotions and Interpersonal Troubles. Educational Academic Mon-thly. 2013; 256(11):88–91. doi: 10.16477/j.cnki.issn1674-2311.2013

[38] Ma HX, Yang SQ, Zhu XD, et al. Relationship between College Students’ Time Management Tendency and Interpersonal Troubles and Mental Healt-h. China Public Health. 2011;27(09):1082–1083.

[39] Xu HY, Yin LT. Investigation and Promotion Strategy of College Students’ Psychological Capital. Journal of Jishou University (Social Science Editio-n). 2018; 39(S1):138–143.

[40] Chen XH. A Survey of Subjective Well-being of College Students in Gene-ral Universities. Journal of National School of Education Administration. 2014(01):78–82.

[41] Zhang PP, Jin XQ. Research on the Relationship between College Students’ Interpersonal Relationship, Psychological Capital and Career Decision-ma-king difficulties. Journal of Anhui Science and Technology University. 2017; 31(05):107–112. doi: 10.19608/j.cnki.1673-8772.2017.0335

[42] Liu HC, Wu MX. Research on the Relationship between Forgiveness, Interpersonal Satisfaction and Subjective Well-being of College Students. Chinese Journal of Clinical Psychology. 2011; 19(04):531–533. doi: 10.16128/j.cnki.1005-3611.2011.04.011

[43] Xiong M, Zhang YH, Ye YD. et al. The Influence of Psychological Capita-l on Adolescents’ Achievement Motivation and Subjective Well-being. Mo-dern Preventive Medicine. 2017; 44(10):1831–1834.

[44] Avey JB, Reichard RJ, Luthans F. et al. Meta-analysis of the Impact of P-ositive Psychological Capital on Employee Attitudes, Behaviors, and Perfor-mance. Human Resource Development Quarterly. 2011, 22: 127–152. doi: 10.1002/hrdq.20070

[45] Tm Guo, Qy Guo, Lb Meng. et al. Research on the Relationship between Psychological Capital and Innovation Performance of Knowledge Workers. Economic Issues. 2019; (10):71–78. doi: 10.16011/j.cnki.jjwt.2019.10.011

[46] Zhang XX. Analysis of the Influence of Psychological Capital on Organiza-tional Performance, Organizational Commitment and Organizational Citize-nship Behavior. Statistics and Decision. 2013(11):108–110. doi: 10.13546/j.cnki.tjyjc.2013.11.051

[47] He B. Research on the Relationship between Psychological Capital, Coping Style and Mental Health of Poor Students in Higher Vocational Colleges. Modern Preventive Medicine. 2014; 41(04):672–675.

[48] Qin JB, Yuan AH. Intervention Effect of Moderate Physical Exercise on College Students’ Inferiority Complex and Psychological Capital. China School Health. 2019; 40(05):756–758. doi: 10.16835/j.cnki.1000-9817.2019.05.030

[49] Huo C, Waheed Akhtar M, Arslan Safdar M, et al. Transformational leader-ship spur organizational commitment among optimistic followers:The role of psychological capital. International Journal of Organizational Leadership.2020; 9(2):93–104. doi: 10.33844/IJOL.2020.60498

[50] Alshahrani ST, Iqbal K. Influence of psychological capital on organizationa-l citizenship behaviors:The mediating role of psychological well-being. Int-ernational Journal of Organizational Leadership.2021; 10(3):299–312. doi: 10.33844/IJOL.2021.60542

